# Mr. Earle in Explanation to Mr. Ramsden

**Published:** 1811-02

**Authors:** Henry Earle

**Affiliations:** Hanover Square


*To the Editors of the Medical and Physical Journal.*

GENTLEMEN,

I SINCERELY regret that the letter which you did me the favour to publish in your last number has considerably offended Mr. Ramsden; it is considered by him as containing animadversions on his practice. In justice, therefore, to myself, and in the hopes of removing all such unpleasant impressions from that Gentleman's mind, I feel myself called upon to state thus publicly, that such never was in the most remote degree my intention.—If that letter admits of any interpretation different from Mr. Ramsden's own account of the operation, I am sorry that I should have adopted expressions capable of a construction which it was not my design they should carry.—By inserting this you will oblige,

Gentlemen,

Your obedient Servant,

HENRY EARLE.

*Hanover Square, Jan. 14th,* 1811.

